# Subcentimetric Papillary Thyroid Carcinoma: Does the Diagnosis Kind Impact Prognosis?

**DOI:** 10.7759/cureus.49563

**Published:** 2023-11-28

**Authors:** Diogo Ramalho, Elisabete Teixeira, Rosa Cueto, Sara Correia, Gustavo Rocha, Maria J Oliveira, Paula Soares, Antonia A Póvoa

**Affiliations:** 1 Endocrinology, Centro Hospitalar de Vila Nova de Gaia/Espinho, Vila Nova de Gaia, PRT; 2 Pathology, Institute of Molecular Pathology and Immunology of the University of Porto (IPATIMUP), Porto, PRT; 3 Pathological Anatomy, Hospital Parc Taulí, Sabadell, ESP; 4 General Surgery, Centro Hospitalar de Vila Nova de Gaia/Espinho, Vila Nova de Gaia, PRT

**Keywords:** disease-free survival, recurrence, prognosis, papillary thyroid microcarcinoma, thyroid neoplasms

## Abstract

Introduction: Subcentimetric papillary thyroid carcinoma (SPTC) (papillary thyroid carcinoma with less than 10 mm in size) usually presents an excellent prognosis, with few aggressive reported cases. Given the globally increased incidence of SPTC, physicians are struggling with the need to identify prognostic factors to stratify SPTC. The aim was to compare clinicopathological variables and prognosis between clinically and incidentally diagnosed SPTC.

Materials and methodsː This is a retrospective observational study on patients with SPTC who underwent thyroidectomy between 2002 and 2015. Two groups were considered: G1 (n=60 (61.9%)), clinical diagnosis (Bethesda III-VI cytology in the thyroid tumor/in cervical lymphadenopathies) and G2 (n=37 (38.1%)), incidental diagnosis (thyroidectomy for benign thyroid pathology). The histological material was reviewed, and molecular analysis of the *BRAF*, *RAS*, and *TERT* promoter (*TERTp*) genes was performed.

Resultsː Ninety-seven individuals were included, 60 (61.9%) of which were from G1, with a predominance of female sex (n=83 (85.6%)). Individuals of G1 were younger (53.0±14.2 versus 59.3±13.9 years; p=0.035), were more frequently treated with 131-iodine (39.2% versus 13.4%; p=0.007), had the largest diameter (8 (p25-p75: 7-9) versus 5 (p25-p75: 4-6.5) mm; p<0.001), and higher frequency of minimal extracapsular invasion (45% versus 24.3%; p=0.041). Increased tumor size was the only independent predictor of a clinical diagnosis (p<0.001).

Conclusionsː Clinically and incidentally diagnosed SPTC showed excellent medium- to long-term prognosis. A larger SPTC was more likely a driver of clinical detection than a marker of tumor aggressiveness, but caution should be taken as contradictory data persists.

## Introduction

In 1989, the World Health Organization (WHO) introduced the term “papillary thyroid microcarcinoma” for papillary thyroid carcinomas (PTC) that measure a maximum of 10 mm in size and later redefined it as subcentimetric papillary thyroid carcinoma (SPTC) [1].

The incidence of SPTC has been increasing widely in the last decade, mainly at the expense of its incidental detection, in histopathological samples of patients submitted to thyroidectomy for presumably benign thyroid conditions [2,3]. The widespread use of high-resolution neck ultrasound has also contributed to the rise of clinical diagnosis of SPTC, due to increased detection of small thyroid nodules, greater accuracy in assessing malignant suspicious ultrasound features, and more optimized guidance of fine needle aspiration biopsy (FNAB), which enhances its diagnostic sensitivity [4]. SPTC accounts for ≥50% of thyroid malignancies [5]. Some authors consider the term “carcinoma” as too disproportionate to SPTC that present usually an excellent prognosis, with rare aggressive reported cases, inducing unnecessary anxiety in patients and families [6,7]. Regarding SPTC prognosis, Choi et al. [8] stated that there were no clinical, biochemical, or pathological factors that easily discriminate indolent from aggressive SPTC, while other authors converge on affirming clinical lymph node metastasis (LNM) at diagnosis as a poor prognostic factor [9-11].

Given the global increase in SPTC incidence, thyroidologists and oncologists are struggling with the need to identify prognostic factors to stratify SPTC. Depending on the type of diagnosis (incidental or clinical), the therapeutic approach of SPTC may range from active surveillance to surgical intervention, which may impact the natural history of SPTC (persistence, recurrence, and disease-related mortality).

The aim of this study was to compare clinicopathological variables and medium- to long-term prognosis between clinically and incidentally diagnosed SPTC.

## Materials and methods

Patient cohort and tissue samples

An observational retrospective study was performed by reviewing electronic records for the attainment of clinical and demographic data of 1,652 consecutive individuals submitted to thyroid surgery at Centro Hospitalar de Vila Nova de Gaia/Espinho between 2002 and 2015. Thyroid cancer was diagnosed in 461 (27.9%) individuals. From these, 97 (21%) were confirmed as SPTC on histopathological analysis and followed at endocrinology consultation. Inclusion criteria were the diagnosis of SPTC in patients older than 18 years, followed for a minimum of two years (unless recurrence or disease-specific mortality occurred earlier), in which there were thyroid specimens for histological re-evaluation.

Formalin-fixed paraffin-embedded (FFPE) material was collected, and all tumor samples were reviewed by a single pathologist (MRB) according to the WHO classification, fourth edition [12]. All doubtful tumor samples were reviewed by two senior pathologists (MSS and MRB) for consensual diagnosis. Each patient was staged according to the eighth edition of the American Joint Committee on Cancer/Union for International Cancer Control (AJCC/UICC) staging system [13].

All procedures described in this study were in accordance with national and institutional ethical standards. The study was conducted in accordance with the Declaration of Helsinki, and the protocol was approved by the local ethics committee (project investigation 30/2016, 2016-01-28, Comissão de Ética do Centro Hospitalar de Vila Nova de Gaia/Espinho). According to Portuguese law, informed consent is not required for retrospective studies.

Patient follow-up

Patients underwent surgery with or without subsequent radioiodine (RAI) treatment. Surgical options depended on thyroid pathology and tumor characteristics (extrathyroidal extension (ETE), clinically identified lymph node metastasis (LNM), and distant metastasis (DM)).

Lymphadenectomy was performed whenever LNMs were found before surgery or intraoperatively. RAI treatment was dependent on the type of surgery performed (total versus lobectomy and isthmectomy and surgery completeness), presence of DM, and post-surgery pathological findings (ETE, lymph vessel and venous invasion, and LNM).

RAI therapy was given four weeks after levothyroxine (LT4) withdrawal or using recombinant thyroid-stimulating hormone (rhTSH) preparation. This treatment was followed by post-therapy scintigraphy, seven days later. Stimulated thyroglobulin (sTg) was evaluated at the time of RAI therapy, and it was defined as the thyroglobulin (Tg) level measured when serum TSH was >30 mIU/L. The post-ablation Tg level was assessed approximately 12 months after surgery. During the first follow-up year, patients were followed every three months and, in subsequent years, twice a year. Serum biochemical evaluation (TSH, free T4, Tg, and anti-Tg antibodies (TgAb)) and neck ultrasonography (US) were routinely performed. Computed tomography (CT) scans, 18-fluorodeoxyglucose positron emission tomography (18-FDG-PET) scans, or RAI thyroid scintigraphy were performed only if previous examinations were doubtful or suspicious. During follow-up, we used the 2015 American Thyroid Association (ATA) risk stratification system [14] to predict the risk of recurrent or persistent disease in patients who were treated with RAI. In non-RAI-treated patients, we used the risk stratification system for patients treated without RAI published by Momesso et al. [15]. All patients were stratified as having four levels of possible response to treatment: excellent, indeterminate, biochemically incomplete, and structurally incomplete.

Structural disease status was detected by appropriate imaging modalities and, whenever possible, confirmed by cytology. Additional surgery was performed whenever feasible. If disease was unresectable, patients received RAI therapy.

Two groups were considered, according to the type of SPTC diagnosis: clinical diagnosis, if thyroid nodule or cervical lymphadenopathy cytology was diagnosed or suggested (Bethesda III-VI) malignancy (G1, n=60 (61.9%)), and incidental diagnosis, if SPTC was diagnosed in thyroid specimens removed for benign thyroid disease (G2, n=37 (38.1%)).

Clinical endpoints

The following clinical and demographic data were evaluated: age at diagnosis, sex, family history of thyroid cancer, thyroid-stimulating hormone (TSH) level at diagnosis of SPTC, thyroid function status (euthyroid, hypothyroidism, and thyrotoxicosis), type of thyroid surgery (total thyroidectomy and hemithyroidectomy), cervical lymphadenectomy, SPTC staging according to the eighth edition of the AJCC/UICC staging system (I, II, and IVb) [13], largest tumor size, focality (unifocal and multifocal), laterality (unilateral and bilateral), histological subtype, major extrathyroidal invasion, minimal ETE (mETE), surgical resection margins (R0 and ≥R1), lymph node metastases and cervical location (central and lateral compartments), lymphocytic thyroiditis, RAI therapy, mutations in the *TERT* (*TERT* promoter (*TERTp*)), *BRAF* (*BRAF*V600E) and *RAS* genes, persistent and/or recurrent disease, disease-free survival period, and all-cause and SPTC-related mortality.

Persistence was defined as any clinical, biochemical, and/or radiological (cervical ultrasound, computed tomography (CT), scintigraphy with 131-Iodine, or positron emission tomography-CT) suspicion or evidence of malignancy (indeterminate or incomplete response to treatment), and recurrence as any evidence of disease after a disease-free period (excellent response to treatment). The disease-free survival period extended from the time of thyroidectomy to the persistence and/or recurrence date or to the last consultation date, if no persistence or recurrence event occurred.

DNA extraction

Histological material was reviewed, and molecular analysis of *BRAF*, *RAS*, and *TERT* gene status was performed (partially reported by Póvoa et al. [16]). DNA from FFPE tissues was retrieved from 10 µm sections after hematoxylin and eosin (H&E)-guided careful microdissection. DNA extraction was performed using the GRS Genomic DNA Kit BroadRange (GRiSP Research Solutions, Porto, Portugal) following the manufacturer’s instructions. Quantitative and qualitative analysis of all samples was then performed by spectrophotometry using NanoDrop ND-1000 Spectrophotometer for microvolume UV-Vis measurements (Thermo Scientific, Waltham, MA).

PCR and Sanger sequencing analysis

Genetic characterization of the series of tumors involving *BRAF*, *RAS* (*NRAS*, *HRAS*, and *KRAS*), and *TERTp* mutations was performed as previously reported [17,18]. Primer design was performed accounting for the most frequent regions mutated in PTC, namely, *BRAF* codon 600, *NRAS* codon 61, *HRAS* and *KRAS* codons 12, 13, and 61, and *TERTp*-124, and *TERTp*−146 regions. Amplification of genomic DNA (25-50 ng) was achieved using the QIAGEN multiplex PCR kit (QIAGEN, Hilden, Germany) following the manufacturer’s instructions. The annealing temperature of 61°C was established after protocol optimization for *BRAF*, *NRAS*, and *TERTp* amplification in the same reaction. *HRAS* and *KRAS* were screened separately by a touchdown PCR using the MyTaq HS Mix 2× Bioline PCR Kit (Meridian Bioscience, Cincinnati, OH) following the manufacturer’s instructions.

PCR amplification was confirmed in 1%-2% agarose gel electrophoresis (GRS Agarose LE, GRiSP, Oporto, Portugal) followed by PCR product purification. All tested hotspot mutations were sequenced using the ABI Prism Big Dye Terminator kit v3.1 Cycle Sequencing (Fisher Scientific Applied Biosystems®, Portsmouth, NH). After sequencing product precipitation, fragments were analyzed by capillary electrophoresis using the Applied Biosystems 3130/3130 ×l Genetic Analyzers (Foster City, CA). For all genes, all detected mutations were validated by performing a new and independent analysis.

Statistical analysis

The Kolmogorov-Smirnov test was used to assess the normality of quantitative variables. Age at diagnosis of SPTC and the disease-free survival period were described as means and standard deviations (SD). The remaining descriptive statistics for continuous variables were reported using medians (first quartile and third quartile) as they were not normally distributed. Categorical variables were expressed as absolute and relative proportions. Univariate analysis was performed to compare variables among the two groups using Fisher’s exact test, unpaired Student’s t-test, and Mann-Whitney U test when they were categorical, normally, and non-normally distributed continuous variables, respectively.

A multivariate logistic regression model was conducted to assess the influence of SPTC-related clinical and sociodemographic factors that were statistically significant in the univariate analysis on the clinical diagnosis of SPTC.

Receiver operating characteristic (ROC) curves were designed to determine the most accurate cut-off values of continuous independent predictors for the clinical diagnosis of SPTC.

Statistical Package for the Social Sciences (SPSS) software (version 27.0) (IBM SPSS Statistics, Armonk, NY) [19] was used, and p<0.05 was considered statistically significant.

## Results

A total of 97 patients were included, with 60 (61.9%) diagnosed clinically (G1) and 37 (38.1%) identified incidentally (G2). Female sex was predominant (n=83 (85.6%)), and the mean age at diagnosis was 55.4±14.3 years. Patients of G1 were younger (p=0.035), were more frequently treated with RAI (p=0.007), had bigger tumors (p<0.001), and had a higher frequency of mETE (p=0.041) (Table 1).

**Table 1:** Univariate analysis stratified by the type of subcentimetric papillary thyroid carcinoma diagnosis. SPTC: subcentimetric papillary thyroid carcinoma, SD: standard deviation, CD: clinical diagnosis, I: incidental diagnosis, TSH: thyroid-stimulating hormone, AJCC: American Joint Committee on Cancer *Statistically significant ^¥^24 (24.7%) cases of omitted data

Variables	CD (n=60 (61.9%))	I (n=37 (38.1%))	p
Clinical and sociodemographic			
Age at diagnosis (years) (mean±SD)	53.0±14.2	59.3±13.9	0.035*
Age at diagnosis (years) (number (%))			0.185
<55	31 (51.7)	14 (37.8)	
≥55	29 (48.3)	23 (62.2)	
Sex (number (%))			0.695
Male	8 (13.3)	6 (16.2)	
Female	52 (86.7)	31 (83.8)	
Family history of thyroid cancer (number (%))			0.796
Yes	7 (11.7)	5 (13.5)	
No	14 (23.3)	12 (32.4)	
Unknown	39 (65)	20 (54.1)	
TSH (μIU/mL) at diagnosis (median (p25, p75))^¥^	0.98 (0.23, 4.22)	2.51 (0.29, 5.68)	0.069
Thyroid function status (number (%))^¥^			
Euthyroidism	14 (23.3)	7 (18.9)	0.903
Hypothyroidism	13 (21.7)	12 (32.4)	0.281
Thyrotoxicosis	21 (35)	6 (16.2)	0.057
Staging and treatments			
131-iodine therapy (number (%))	38 (63.3)	13 (35.1)	0.007*
Surgery (number (%))			0.133
Total thyroidectomy	56 (93.3)	31 (83.8)	
Hemithyroidectomy	4 (6.7)	6 (16.2)	
Cervical lymphadenectomy (number (%))	6 (10)	1 (2.7)	0.177
Stage (AJCC eighth edition^[13]^) (number (%))			0.618
I	58 (96.6)	35 (94.6)	
II	1 (1.7)	2 (5.4)	
IVB	1 (1.7)	0 (0)	
Histopathological			
Largest tumor size (mm) (median (p25-p75))	8 (7-9)	5 (4-6.5)	<0.001*
Focality (number (%))			0.082
Unifocal	35 (58.3)	28 (75.7)	
Multifocal	25 (41.7)	9 (24.3)	
Laterality (number (%))			0.229
Unilateral	50 (83.3)	34 (91.9)	
Bilateral	10 (16.7)	3 (8.1)	
Histological subtype (number (%))			0.124
Classical	47 (78.2)	20 (54.1)	
Follicular	10 (16.7)	13 (35.1)	
Tall cell	1 (1.7)	1 (2.7)	
Oncocytic	1 (1.7)	3 (8.1)	
Warthin-like	1 (1.7)	0 (0)	
Major extrathyroidal extension (number (%))	1 (1.7)	0 (0)	0.430
Minimal extrathyroidal extension (number (%))	27 (45)	9 (24.3)	0.041*
Surgical resection margins (number (%))			0.117
R0	54 (90)	36 (97.3)	
≥R1	6 (10)	1 (2.7)	
Lymph node metastases (number (%))	7 (11.7)	2 (5.4)	0.276
Central compartment	3 (5)	2 (5.4)	
Lateral compartment	4 (6.7)	0 (0)	
Lymphocytic thyroiditis (number (%))	26 (43.3)	13 (35.1)	0.424
Molecular (mutations)			
TERTp (number (%))	4 (7.4)	0 (0)	0.115
*BRAF*^V600E^ (number (%))	40 (69)	23 (63.9)	0.611
TERTp and *BRAF*^V600E^ (number (%))	3 (5)	0 (0)	0.172
Prognosis			
Persistence (number (%))	8 (13.3)	8 (21.6)	0.285
Recurrence (number (%))	5 (8.3)	0 (0)	0.071
Disease-free survival period (months) (mean±SD)	70.7±33.6	62.0±40.0	0.797
All-cause mortality (number (%))	3 (5)	3 (8.1)	0.537
Attributable to SPTC	2 (3.3)	0 (0)	0.262

G1 also showed a tendentially higher frequency of multifocality (G1: 41.7% versus G2: 24.3%; p=0.082), bilaterality (G1: 16.7% versus G2: 8.1%; p=0.229), positive surgical resection margins ≥ R1 (G1: 10% versus G2: 2.7%; p=0.117), lymph node metastasis (G1: 11.7% versus G2: 5.4%; p=0.276), and *TERTp* mutations (G1: 7.4% versus G2: 0%; p=0.115), although without statistical significance.

During the follow-up period (67.4±36.2 months), SPTC persistence rates and mean disease-free survival periods remained comparable between groups, although G1 evidenced a recurrence rate of 8.3% and G2 reported no cases of recurrence (p=0.071). Six (6.2%) patients died; in two (2.1%) cases, mortality was SPTC-attributable and were all clinically detected. No cases of SPTC-specific mortality were reported in patients with an incidental diagnosis. SPTC-specific mortality was tendentially higher in the clinically diagnosed group, although no statistical significance was found (p=0.262).

No statistically significant differences were reported between groups for the presence of thyroid cancer family history, TSH level at SPTC diagnosis, thyroid function status, type of thyroid surgery, presence of cervical lymphadenectomy, SPTC staging, SPTC histological subtype, presence of major ETE invasion, lymphocytic thyroiditis, and *BRAF* mutation status (Table 1). No *RAS* gene mutations were identified.

The multivariate logistic regression model showed that increased tumor size was the only independent factor predictive of a clinical diagnosis of SPTC (odds ratio (OR): 1.785, 95% confidence interval (CI): 1.368-2.329; p<0.001) (Table 2).

**Table 2:** Logistic regression of variables predictive of a clinical diagnosis of subcentimetric papillary thyroid carcinoma. β: adjusted regression coefficient, SE: standard error, OR: odds ratio, 95% CI: 95% confidence interval *Statistically significant

Variables	β	SE	OR (95% CI)	p
Age at diagnosis (+1 year)	0.028	0.019	1.029 (0.990-1.069)	0.145
131-iodine therapy (yes)	0.829	0.530	2.291 (0.811-6.473)	0.118
Largest tumor size (+1 mm)	1.760	0.136	1.785 (1.368-2.329)	<0.001*
Minimal extrathyroidal extension (yes)	0.001	0.591	1.001 (0.314-3.189)	0.999

**Figure 1:**
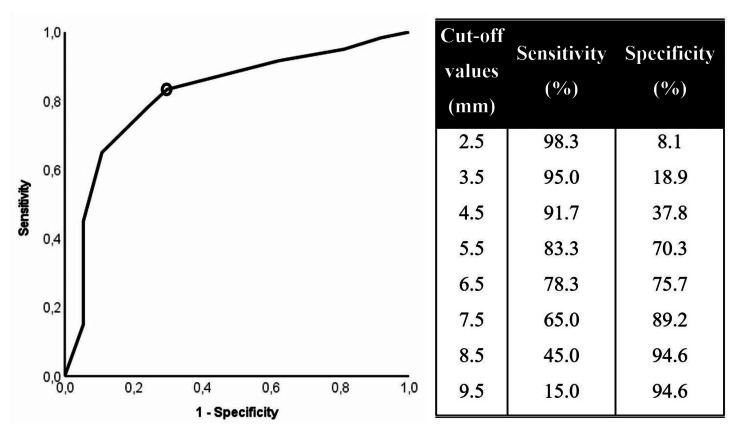
Receiver operating characteristic curve analysis for the clinical diagnosis of subcentimetric papillary thyroid carcinoma. AUC: 0.820 (95% CI: 0.73000.910; p<0.001) AUC: area under the curve, CI: confidence interval The black circle indicates the most accurate cut-off value (5.5 mm).

After ROC curve analysis, a tumor size of 5.5 mm was the most accurate cut-off value (sensitivity: 83.3%, specificity: 70.3%), demonstrating the power to discriminate clinically diagnosed SPTC (area under the curve (AUC): 0.820, 95% CI: 0.730-0.910, p<0.001) (Figure 1).

To assess variables of interest related to an SPTC size ≥ 5.5 mm, a subanalysis was performed. In addition to clinically diagnosed SPTC, RAI therapy, total thyroidectomy, and mETE were significantly associated with a tumor size of ≥5.5 mm. LNM rates and prognostic variables were similar between groups (Table 3).

**Table 3:** Univariate analysis stratified by subcentimetric papillary thyroid carcinoma size. SPTC: subcentimetric papillary thyroid carcinoma, SD: standard deviation, TSH: thyroid-stimulating hormone, AJCC: American Joint Committee on Cancer *Statistically significant ^¥^24 (24.7%) cases of omitted data

Variables	<5.5 mm (n=36 (37.1%))	≥5.5 mm (n=61 (62.9%))	p
Clinical and sociodemographic			
Age at diagnosis (years) (mean±SD)	57.4±14.1	54.2±14.4	0.294
Age at diagnosis (years) (number (%))			0.473
<55	15 (41.7)	30 (49.2)	
≥55	21 (58.3)	31 (50.8)	
Sex (number (%))			0.281
Male	7 (19.4)	7 (11.5)	
Female	29 (80.6)	54 (88.5)	
Family history of thyroid cancer (number (%))			0.658
Yes	6 (16.7)	6 (9.8)	
No	11 (30.5)	15 (24.6)	
Unknown	19 (52.8)	40 (65.6)	
TSH (μIU/mL) at diagnosis (median (p25, p75))^¥^	2.01 (0.20, 8.18)	0.41 (0.05, 4.26)	0.176
Thyroid function status (number (%))^¥^			
Euthyroidism	7 (19.4)	13 (21.3)	0.976
Hypothyroidism	11 (30.6)	14 (23)	0.228
Thyrotoxicosis	7 (19.4)	20 (32.8)	0.225
Staging and treatments			
131-iodine therapy (number (%))	14 (38.9)	37 (60.7)	0.038*
Surgery (number (%))			0.023*
Total thyroidectomy	29 (80.6)	58 (95.1)	
Hemithyroidectomy	7 (19.4)	3 (4.9)	
Cervical lymphadenectomy (number (%))	3 (8.3)	4 (6.6)	0.744
Stage (AJCC eighth edition^[13]^) (number (%))			0.586
I	34 (94.4)	59 (96.7)	
II	2 (5.6)	2 (3.3)	
IVB	0 (0)	0 (0)	
Type of diagnosis (number (%))			<0.001*
Clinical	10 (27.8)	50 (82)	
Incidental	26 (72.2)	11 (18)	
Histopathological			
Focality (number (%))			0.476
Unifocal	25 (69.4)	38 (62.3)	
Multifocal	11 (30.6)	23 (37.7)	
Laterality (number (%))			0.260
Unilateral	33 (91.7)	51 (83.6)	
Bilateral	3 (8.3)	10 (16.4)	
Histological subtype (number (%))			0.483
Classical	22 (61.1)	45 (73.8)	
Follicular	10 (27.7)	13 (21.4)	
Tall cell	1 (2.8)	1 (1.6)	
Oncocytic	3 (8.4)	1 (1.6)	
Warthin-like	0 (0)	1 (1.6)	
Major extrathyroidal extension (number (%))	0 (0)	1 (1.6)	0.440
Minimal extrathyroidal extension (number (%))	6 (16.7)	30 (49.2)	0.001*
Surgical resection margins (number (%))			0.135
R0	36 (100)	54 (88.5)	
≥R1	0 (0)	7 (11.5)	
Lymph node metastases (number (%))			0.501
Central compartment	2 (5.6)	2 (3.3)	
Lateral compartment	1 (2.8)	5 (8.2)	
Lymphocytic thyroiditis (number (%))	15 (38.5)	24 (39.3)	0.822
Molecular (mutations)			
*TERTp* (number (%))	0 (0)	4 (7.1)	0.134
*BRAF*^V600E^ (number (%))	19 (63.3)	35 (67.3)	0.715
*TERTp* and *BRAF*^V600E^ (number (%))	0 (0)	3 (4.9)	0.194
Prognosis			
Persistence (number (%))	8 (22.2)	8 (13.1)	0.243
Recurrence (number (%))	2 (5.6)	3 (4.9)	0.891
Disease-free survival period (months) (mean±SD)	61.7±36.9	70.7±35.7	0.238
All-cause mortality (number (%))	3 (8.3)	3 (4.9)	0.500
Attributable to SPTC	1 (2.8)	1 (1.6)	0.703

In the multivariate analysis, clinically diagnosed SPTC and mETE were independently associated with an SPTC size of ≥5.5 mm (Table 4).

**Table 4:** Logistic regression of variables predictive of a subcentimetric papillary thyroid carcinoma size of ≥5.5 mm. β: adjusted regression coefficient, SE: standard error, OR: odds ratio, 95% CI: 95% confidence interval *Statistically significant **Reference category: hemithyroidectomy ***Reference category: incidental diagnosis

Variables	β	SE	OR (95% CI)	p
131-iodine therapy (yes)	-0.088	0.576	0.916 (0.296-2.832)	0.879
Total thyroidectomy (yes)**	1.315	0.923	3.726 (0.610-22.768)	0.154
Minimal extrathyroidal extension (yes)	1.423	0.601	4.152 (1.277-13.492)	0.018*
Clinical diagnosis (yes)***	2.390	0.545	10.919 (3.750-31.788)	<0.001*

## Discussion

In our study, larger tumor size was the only independent factor for a clinical diagnosis of SPTC, specifically in tumors ≥ 5.5 mm. This observation is concordant with most published evidence [20-23], although some authors did not find any association [24,25].

Our subanalysis showed an independent association between mETE and a larger SPTC size (≥5.5 mm), which is supported by previous findings [26-28]. SPTC size did not affect the presence of LNM and prognosis (Table 3), contradicting the most recognizable evidence as published by other authors, at least regarding LNM, persistence/recurrence risk, and disease-free survival [28-32]. However, these studies [28-32] included all sizes of PTC and not exclusively SPTC. The eighth edition of the AJCC/UICC staging system [13] and the ATA 2015 risk stratification system [14] also do not provide specific guidance on subdifferentiation by tumor size for SPTC, focusing solely on discriminating tumor size cut-off values above 1 cm (i.e., 1, 2, and 4 cm). Therefore, we highlight the need for larger studies on SPTC to accurately assess the impact of their size on prognosis, especially regarding lymph node metastasis, persistence/recurrence, and disease-free survival.

Patients with clinically diagnosed SPTC were younger, were more often submitted to RAI therapy, and had a higher frequency of mETE. However, these associations were observed merely in the univariate analysis (Table 1), concordant with previous analyses. In addition to a heightened propensity for highly aggressive tumor behavior in earlier age cohorts, younger patients exhibited a proclivity for increased medical scrutiny and more frequent utilization of fine needle aspiration biopsy (FNAB), thereby providing a potential rationale for its correlation with a clinical diagnosis [23,24]. Once our cohort of patients was mainly treated before 2015, the higher frequency of RAI therapy in both groups might be related to the expansion of the “ATA low risk” category from the 2009 ATA guidelines [33] to the 2015 ATA guidelines (intrathyroidal, papillary microcarcinoma, and unifocal or multifocal, including *BRAF*V600E mutated; intrathyroidal, encapsulated follicular variant of papillary thyroid cancer) [14], which best explains a subsequent decrease in the number of cases with clear indication for RAI treatment, since 2015. Until recently, mETE was considered one prominent independent predictor of recurrence and survival [33,34]. While the 2015 ATA guidelines [14] still define mETE as a predictor of intermediate risk of recurrence, other published data have been divergent in this regard as described below. In fact, the exclusion of mETE from the eighth edition of the AJCC/UICC staging system [13] was predicated upon the absence of substantive evidence of a prognostic impact on survival. mETE weight on disease persistence/recurrence remains a matter of debate [35]. Some studies [35-37] found an association between mETE and increased overall recurrence risk, but the meta-analysis [37] included studies with divergent findings in this matter, which ultimately resulted in a nonsignificant small increase in absolute risk of recurrence (OR: 2.40, 95% CI: 0.95-6.03). Evidence is more consistent in PTC without LNM, as several studies found no association between mETE and recurrence risk [38,39]. Of note, the majority of these studies did not include histopathological subtypes and/or the presence of vascular invasion, which are major recurrence risk factors. Hence, putting together the evidence highlighted above, we can advance that isolated mETE might not add substantial risk to global survival and recurrence of SPTC without LNM, but its actual effect on the recurrence risk of SPTC with LNM remains unclear.

In our study, clinically diagnosed SPTC is more frequently associated with other histological and molecular markers of aggressive behavior (multifocality, bilaterality, incomplete surgical resection margins, lymph node metastasis, and *TERTp* mutations), recurrence, and SPTC-specific mortality. Although these associations did not reach statistical significance, they are supported by robust evidence from previous studies [40,41]. A tendency of association was found for multifocality and clinically diagnosed SPTC, but it seems to be better explained by the greater ease in obtaining a clinical diagnosis with a higher number of SPTC foci than an association with greater tumor aggressiveness, as outlined by other reports [20,24,42]. Other studies stated that multifocality was not a predictive factor of a clinical diagnosis [21,22,43]. The ATA 2015 risk stratification system [14] classifies a multifocal SPTC as possessing an “intermediate risk” of recurrence (5%-20%) only if an extrathyroidal extension is concomitantly present. Accordingly, intrathyroidal SPTC is categorized as “low risk” of recurrence (<5%), independently of focality [14]. The eighth edition of the AJCC/UICC staging system [13] considers four independent prognostic factors of SPTC, namely, age, sex, tumor size, and LNM, and thus, focality is not included in the staging system. A higher frequency of LNM has been consistently reported in clinically diagnosed SPTC. Once FNAB is rarely indicated in thyroid micronodules, clinical cases were frequently diagnosed by FNAB assessment of adenopathy, which largely explains the association between a clinical diagnosis of SPTC and the presence of LNM [20-22,24,25,42]. Conversely, *TERTp* mutations have been related to more aggressive behavior of SPTC, and consequently, poor prognostic outcomes have been reported in such patients [44].

Therefore, taking those analyses into account, the association of larger tumor size with clinical diagnosis seems to be best explained by a greater ability to detect larger lesions through imaging techniques than the presence of greater intrinsic tumor aggressiveness. However, two older studies found an association between larger tumor size and greater aggressive behavior of SPTC, and thus, this needs to be clarified by more robust evidence [40,41].

Clinically diagnosed patients underwent FNAB and subsequently proceeded to surgery with a median largest tumor size of 8 mm. This decision is supported by the fact that the majority of these patients were young adults with nodules categorized as European Thyroid Imaging Reporting and Data System (EU-TIRADS) 5, nearing the threshold for FNAB, or presenting locoregional adenopathies. Despite the consistent option of active surveillance, the choice of surgical intervention was guided by the patient’s personal preference. This decision was influenced by a desire to alleviate anxiety associated with maintaining active surveillance in the context of a known malignancy.

In our analysis, both groups demonstrated reasonable persistence and recurrence rates and extended mean disease-free survival time, similar to the mean follow-up period of around five years and a half. A nonsignificant higher recurrence rate was observed in clinically diagnosed SPTC when compared to incidentally diagnosed cases, which did not report any recurrence (G1: 8.3% versus G2: 0%; p=0.071). Published series are divergent regarding this topic (association [45,46] versus no association [21,23,42]).

Despite the reasonable persistence and recurrence rates and the potential link to histopathological and molecular markers of aggressive behavior, medium- to long-term survival prognosis was excellent in clinically diagnosed SPTC, as our study revealed only two (3.3%) deaths attributable to SPTC in clinically diagnosed cases, which is comparable to the low SPTC-related mortality rates reported in the literature for both clinically and incidentally diagnosed cases (i.e., up to 3.8% and 2.4%, respectively) [22,43]. Both patients who had disease-related death were elderly and presented with lateral compartment LNM.

However, there are some limitations that require particular attention. First, the retrospective nature of our approach, reliant on electronic records from 2002 to 2015, introduces inherent challenges, including susceptibility to selection bias, incomplete data, and the complexity of establishing causation. Second, the study’s single-center focus raises concerns about the generalizability of findings, as the patient cohort may not fully represent the diverse demographic and clinical characteristics of a broader population. Third, the relatively small sample size may restrict the statistical power and overall reliability of our study. Larger series are needed to further investigate our findings. Finally, the specified follow-up duration might be insufficient to capture long-term outcomes and survival data, particularly for a cancer type such as SPTC, known for its generally indolent course. A more extended follow-up period would provide a more comprehensive understanding of the disease trajectory. These limitations should be considered when interpreting and applying the findings of our study.

## Conclusions

A larger tumor size was more likely linked to the clinical detection of SPTC, rather than indicating a theoretically higher aggressiveness in these clinically diagnosed cases. While aggressive histopathological and molecular features, as well as recurrence, showed nonsignificant associations with clinically detected SPTC, it is important to note the potential influence of a small sample size bias. Notably, both clinically and incidentally diagnosed cases demonstrated excellent medium- to long-term survival outcomes, characterized by low SPTC-related mortality rates and extended disease-free survival times. However, conflicting evidence persists in the published literature, underscoring the need for additional research, particularly larger prospective studies, to provide more conclusive insights in this domain.
